# Sex differences in fracture outcomes within Taiwan population: A nationwide matched study

**DOI:** 10.1371/journal.pone.0231374

**Published:** 2020-04-09

**Authors:** Fang-Pai Chou, Hung-Chi Chang, Chun-Chieh Yeh, Chih-Hsing Wu, Yih-Giun Cherng, Ta-Liang Chen, Chien-Chang Liao

**Affiliations:** 1 Department of Anesthesiology, Shuang Ho Hospital, Taipei Medical University, New Taipei City, Taiwan; 2 Department of Anesthesiology, School of Medicine, College of Medicine, Taipei Medical University, Taipei, Taiwan; 3 Department of Surgery, China Medical University Hospital, China Medical University, Taichung, Taiwan; 4 Department of Surgery, University of Illinois, Illinois, Chicago, United States of America; 5 Department of Family Medicine, National Cheng Kung University Hospital, Tainan, Taiwan; 6 Department of Anesthesiology, Wan Fang Hospital, Taipei Medical University, Taipei, Taiwan; 7 Anesthesiology and Health Policy Research Center, Taipei Medical University Hospital, Taipei, Taiwan; 8 Department of Anesthesiology, Taipei Medical University Hospital, Taipei, Taiwan; 9 Research Center of Big Data and Meta-Analysis, Wan Fang Hospital, Taipei Medical University, Taipei, Taiwan; 10 School of Chinese Medicine, College of Chinese Medicine, China Medical University, Taichung, Taiwan; National Yang-Ming University, TAIWAN

## Abstract

**Background and aims:**

Because the sex difference in outcomes of fracture was incompletely understood, we evaluated the post-fracture complications and mortality of female and male patients.

**Methods:**

We conducted a nationwide study of 498,586 fracture patients who received inpatient care using Taiwan’s National Health Insurance Research Database 2008–2013 claims data. Female and male fracture patients were selected for comparison by using a propensity-score matching procedure. Age, low income, types of fracture, fracture with surgery, several medical conditions, number of hospitalization and emergency visits were considered as potential confounding factors. Multivariate logistic regressions were used to calculate the adjusted odds ratios (OR), the 95% CI of post-fracture complications and 30-day in-hospital mortality differences between women and men.

**Results:**

Male patients had a higher risk of post-fracture pneumonia (OR 1.96, 95% CI 1.83–2.11), acute renal failure (OR 1.85, 95% CI 1.60–2.15), deep wound infection (OR 1.63, 95% CI 1.51–1.77), stroke (OR 1.58, 95% CI 1.49–1.67), septicemia (OR 1.51, 95% CI 1.42–1.61), acute myocardial infarction (OR 1.38, 95% CI 1.09–1.75) and 30-day in-hospital mortality (OR 1.69, 95% CI 1.48–1.93) compared with female patients. However, a lower risk of post-fracture urinary tract infection (OR 0.69, 95% CI 0.65–0.72) was found in men than in women. Male patients also had longer hospital stays and higher medical expenditures due to fracture admission than did the female patients. Higher rates of post-fracture adverse events in male patients were noted in all age groups and all types of fractures.

**Conclusion:**

We raised the possibility that male patients showed more complications and higher mortality rates after fracture admission compared with female patients, with the exception of urinary tract infections.

## Introduction

Fractures are an important public health problem that affects populations all over the world, especially hip fractures. The annual mean number of hip fractures was 957.3 per 100,000 for women and 414.4 per 100,000 for men in the United States [1]. A typical patient with a hip fracture spends US $40000 in the first year following a hip fracture for direct medical costs and almost $5000 in subsequent years [1]. Mortality, morbidity and a poor recovery after a fracture not only affect a patient’s activities of daily living but also cost a significant amount of money in medical expenses. Therefore, post-fracture outcomes and factors that affect the prognosis are important.

Some studies have found no differences in fracture outcome between genders [2,3]. A Finnish study has reported worse survival after fracture in female patients than in male patients [4]. In contrast, several previous studies have shown gender differences in short-term and long-term mortality, adverse outcomes and post-fracture functional recovery, mostly indicating that men, for unknown reasons, tend to have worse post-fracture outcomes than women [5–13]. However, many of the previous studies were limited by a small sample size [7–9,13], an absence of adequate matching [5,6,8,9,11–13], and by focusing on single type of fracture [5–13] or a specific population [3,5,8–10]. These limitations made the interpretation about the association between gender and outcomes inconclusive and incompletely understood. In addition, limited information was available on the sex differences of fracture outcomes in Taiwan populations.

Using Taiwan's National Health Insurance Research Database, we conducted a nationwide matched cohort study that included all fracture patients from 2008–2013. The aim of our study was to compare the post-fracture complications of women and men.

## Methods

### Source of data

Taiwan’s National Health Insurance program was initiated in March 1995, and it now covers more than 99% of Taiwan’s 23 million residents. In this study, we used reimbursement claims data from Taiwan’s National Health Insurance Research Database, which records patients’ basic characteristics and medical services. This information includes physicians’ primary and secondary diagnoses, treatment, procedures, prescriptions, the medical expenses of outpatient care, emergency care, and hospitalizations [14–16].

The data underlying this study is from the National Health Insurance Research Database, which has been transferred to the Health and Welfare Data Science Center. Interested researchers can obtain the data through formal application to the Health and Welfare Data Science Center, Department of Statistics, Ministry of Health and Welfare, Taiwan (http://dep.mohw.gov.tw/DOS/np-2497-113.html). Under the regulations from the National Health Insurance Research Institutes, we have made the formal application (included application documents, study proposals, and ethics approval of the institutional review board) of the current insurance data from in 2015. The authors of the present study had no special access privileges in accessing the data which other interested researchers would not have.

### Ethics approval

As these reimbursement claims were used in this study, the electronic database was decoded with patient identifications scrambled for further academic access for research to protect privacy. We conducted this study in accordance with the Declaration of Helsinki. Although the National Health Research Institutes exempt such uses from informed consent, because patient identifications are decoded and scrambled, this study was evaluated and approved by the Institutional Review Board of Taipei Medical University (TMU-JIRB-201404070; TMU-JIRB-201505055; TMU-JIRB-201705084).

### Study design

Among the >3 million surgical patients over 20 years of age in Taiwan between 2008 and 2013, we identified 498,586 patients with a fracture-related hospitalization. For the comparison of fracture outcomes between women and men, each female fracture patient was randomly matched to a male fracture patient, using a propensity score matched-pair procedure to balance the differences in baseline characteristics. After propensity score matching (case-control ratio, 1:1), there were 100,864 female patients and 100,864 male patients.

### Measures and definitions

According to the regulations from the Bureau of National Health Insurance in Taiwan, people with low-income status were qualified to have the registration fee and medical copayment waived when they received outpatient, emergency, and inpatient medical care. In this study, low socioeconomic status was defined as a patient who had a record of a waived registration fee and medical copayment due to low income during the 2 years prior to the fracture-related hospitalization. We used *The International Classification of Diseases*, *Ninth Revision*, *Clinical Modification* (ICD-9-CM) and physicians’ diagnoses to identify patients’ history of diseases. Based on our previous studies, pre-fracture medical conditions that were identified via medical claims for the 24-month pre-fracture period included hypertension (ICD-9-CM 401–405), hyperlipidemia (ICD-9-CM 272.0, 272.1, and 272.2), peptic ulcer disease (ICD-9-CM 531–533), diabetes (ICD-9-CM 250), mental disorders (ICD-9-CM 290–319), ischemic heart disease (ICD-9-CM 410–414), atherosclerosis (ICD-9-CM 440), chronic obstructive pulmonary disease (ICD-9- CM 491,492, 496), anemia (ICD-9-CM 280–285), heart failure (ICD-9-CM 428), chronic kidney disease (ICD-9-CM 580–585), cancer (ICD-9-CM 140–208), osteoporosis (ICD-9-CM 733.0), stroke (ICD-9-CM 430–438), and liver cirrhosis (ICD-9-CM 571.2, 571.5, 571.6). Renal dialysis was identified by an administration code (D8, D9). Nine major in-hospital complications after fracture were noted, including pulmonary embolism (ICD-9-CM 415), stroke (ICD-9-CM 430–438), pneumonia (ICD-9-CM 480–486), urinary tract infection (ICD-9-CM 599.0), septicemia (ICD-9-CM 038, 998.5), acute renal failure (ICD-9-CM584), postoperative bleeding (ICD-9-CM 998.0, 998.1 and 998.2), acute myocardial infarction (ICD-9-CM 410), and deep wound infection (ICD-9-CM 958.3). Length of hospital stay and medical expenditures after the initial fracture admission were analyzed as secondary outcomes, and differences were noted between female and male patients. The 30-day in-hospital mortality is the main outcome of this study. Fracture patient’s medical expenditure was calculated by accounting all National Health Insurance payment during the index fracture hospitalization included surgical procedures, medications, admission stay, and materials. However, self-payment expenditure (out-off pocket money) was not included in this study.

### Statistical analysis

To reduce confounding errors, this study used a propensity score-matched pair procedure to balance the covariates between female fracture patients and male fracture patients. We developed a non-parsimonious multivariable logistic regression model to estimate a propensity score for female and male fracture patients. Clinical significance guided the initial choice of covariates in this model. Covariates included age, low income, medical condition, and type of fracture. A structured iterative approach was used to refine this model with the goal of achieving a covariate balance within the matched pairs. We used chi-square tests to measure covariate balance, and p < 0.05 was used to represent a meaningful covariate imbalance. We matched female patients to male patients using a greedy matching algorithm with a caliper width of 0.2 standard deviation of the log odds of the estimated propensity score [17–19].

Adjusted odds ratios (OR) with 95% confidence intervals (CI) for 30-day complications and mortality after fracture between female and male patients were calculated in the multivariate logistic regression models by adjusting for age, low-income, medical conditions, and type of fracture. We also performed stratification analysis by age, coexisting medical conditions, and type of fracture for the risk of post-fracture adverse events (including 30-day in-hospital mortality, stroke, pneumonia, septicemia, acute renal failure, acute myocardial infarction, and deep wound infection) associated with sex. SAS version 9.1 (SAS Institute Inc., Cary, NC) statistical software was used for data analyses; 2-sided p < 0.05 indicated statistically significant differences.

## Results

Before matching with propensity scores (Table 1), male fracture patients had higher proportions of young age of 20–29 years (p < 0.0001), low income (p < 0.0001), skull bone fracture (p < 0.0001), chronic obstructive pulmonary disease (p < 0.0001), and liver cirrhosis (p < 0.0001). However, male fracture patients had lower proportions of hypertension (p < 0.0001), hyperlipidemia (p < 0.0001), peptic ulcer disease (p < 0.0001), diabetes (p < 0.0001), mental disorders (p < 0.0001), ischemic heart disease (p < 0.0001), atherosclerosis (p < 0.0001), anemia (p < 0.0001), heart failure (p < 0.0001), and renal dialysis (p < 0.0001). After matching with propensity scores (Table 2), there were no differences in age, medical conditions, fracture type, and history of emergency care and hospitalization between female and male patients.

**Table 1:** Baseline characteristics in female and male fracture patients before matching.

	Female (n = 249012)		Male (n = 249574)		p
Age, years	n	(%)	n	(%)	<0.0001
20–29	18582	(7.5)	44607	(17.9)	
30–39	17066	(6.9)	40456	(16.2)	
40–49	23655	(9.5)	43903	(17.6)	
50–59	46832	(18.8)	40764	(16.3)	
60–69	45149	(18.1)	26091	(10.5)	
70–79	52236	(21.0)	26683	(10.7)	
≥80	45492	(18.3)	27070	(10.9)	
Low income					<0.0001
No	243314	(97.7)	241805	(96.9)	
Yes	5698	(2.3)	7769	(3.1)	
Types of fracture					<0.0001
Skull	2509	(1.0)	8027	(3.2)	
Face bones	7493	(3.0)	13988	(5.6)	
Unqualified skull fracture	618	(0.3)	2204	(0.9)	
Vertebral column	13445	(5.4)	9973	(4.0)	
Rib, sternum, larynx or trachea	1976	(0.8)	4539	(1.8)	
Pelvis	1729	(0.7)	2258	(0.9)	
Other ill-defined fractures of trunk	3	(0.0)	1	(0.0)	
Clavicle or scapula	14061	(5.7)	25743	(10.3)	
Humerus, radius or ulna	76446	(30.7)	48697	(19.5)	
Carpal, metacarpal, or phalanges	7550	(3.0)	21041	(8.4)	
Other upper limb	3	(0.0)	9	(0.0)	
Fracture of femur	67951	(27.3)	46733	(18.7)	
Fracture of patella, tibia, or fibula	27603	(11.1)	31760	(12.7)	
Ankle, tarsal, metatarsal bones, phalanges of foot	27424	(11.0)	34217	(13.7)	
Other lower limb	26	(0.01)	36	(0.01)	
Multiple fractures of upper limbs, lower limbs or trunk	174	(0.1)	342	(0.1)	
Fracture of unspecified bones	1	(0.0)	6	(0.0)	
Fracture with surgery	176588	(70.9)	185034	(74.1)	<0.0001
Medical conditions					
Hypertension	116198	(46.7)	74303	(29.8)	<0.0001
Mental disorders	50508	(20.3)	35379	(14.2)	<0.0001
Diabetes	56162	(22.6)	33988	(13.6)	<0.0001
Hyperlipidemia	56317	(22.6)	31595	(12.7)	<0.0001
Peptic ulcer disease	27841	(11.2)	21716	(8.7)	<0.0001
COPD	15387	(6.2)	20760	(8.3)	<0.0001
Ischemic heart disease	25223	(10.1)	17374	(7.0)	<0.0001
Atherosclerosis	18271	(7.3)	12759	(5.1)	<0.0001
Anemia	19319	(7.8)	10117	(4.1)	<0.0001
Heart failure	11142	(4.5)	6421	(2.6)	<0.0001
Liver cirrhosis	2203	(0.9)	3553	(1.4)	<0.0001
Renal dialysis	4310	(1.7)	2919	(1.2)	<0.0001
Chronic kidney disease	6736	(2.7)	5179	(2.1)	<0.0001
Asthma	13050	(5.2)	8763	(3.5)	<0.0001
Stroke	7505	(3.0)	6474	(2.6)	<0.0001
Cancer	12231	(4.9)	9184	(3.7)	<0.0001
Osteoporosis	13280	(5.3)	2756	(1.1)	<0.0001
Alcohol-related illness	1773	(0.7)	9390	(3.8)	<0.0001
Parkinson's disease	6616	(2.7)	4297	(1.7)	<0.0001
Number of hospitalizations*					<0.0001
0	204202	(82.0)	208629	(83.6)	
1	29467	(11.8)	25947	(10.4)	
2	8674	(3.5)	7967	(3.2)	
≥3	6669	(2.7)	7031	(2.8)	
Number of emergency visits*					<0.0001
0	147898	(59.4)	145488	(58.3)	
1	61665	(24.8)	63513	(25.5)	
2	21878	(8.8)	23055	(9.2)	
≥3	17571	(7.1)	17518	(7.0)	

COPD, chronic obstructive pulmonary disease.

*Before fracture admission within one year

**Table 2:** Baseline characteristics in female and male fracture patients after matching.

	Female (n = 100864)		Male (n = 100864)	
Age, years	n	(%)	n	(%)
20–29	14965	(14.8)	14965	(14.8)
30–39	12206	(12.1)	12206	(12.1)
40–49	15635	(15.5)	15635	(15.5)
50–59	24800	(24.6)	24800	(24.6)
60–69	12237	(12.1)	12237	(12.1)
70–79	10112	(10.0)	10112	(10.0)
≥80	10909	(10.8)	10909	(10.8)
Low income				
No	99769	(98.9)	99769	(98.9)
Yes	1095	(1.1)	1095	(1.1)
Types of fracture*				
Skull	1391	(1.4)	1391	(1.4)
Face bones	4872	(4.8)	4872	(4.8)
Unqualified skull fracture	281	(0.3)	281	(0.3)
Vertebral column	3093	(3.1)	3093	(3.1)
Rib, sternum, larynx or trachea	684	(0.7)	684	(0.7)
Pelvis	544	(0.5)	544	(0.5)
Clavicle or scapula	9420	(9.3)	9420	(9.3)
Humerus, radius or ulna	25991	(25.8)	25991	(25.8)
Carpal, metacarpal, or phalanges	4962	(4.92)	4962	(4.9)
Other upper limb	0	(0.0)	0	(0.0)
Fracture of femur	19281	(19.1)	19281	(19.1)
Fracture of patella, tibia, or fibula	14702	(14.6)	14702	(14.6)
Ankle, tarsal, metatarsal bones, phalanges of foot	15583	(15.5)	15583	(15.5)
Other lower limb	1	(0.0)	1	(0.0)
Multiple fractures of upper limbs, lower limbs or trunk	59	(0.1)	59	(0.1)
Fracture with surgery	89500	(78.8)	89500	(78.8)
Medical conditions				
Hypertension	33221	(29.2)	33221	(29.2)
Diabetes	13693	(12.1)	13693	(12.1)
Hyperlipidemia	13863	(12.2)	13863	(12.2)
Mental disorders	12459	(11.0)	12459	(11.0)
Peptic ulcer disease	6226	(5.5)	6226	(5.5)
Ischemic heart disease	3987	(3.5)	3987	(3.5)
COPD	3436	(3.0)	3436	(3.0)
Atherosclerosis	2607	(2.3)	2607	(2.3)
Anemia	2008	(1.8)	2008	(1.8)
Heart failure	881	(0.8)	881	(0.8)
Renal dialysis	237	(0.2)	237	(0.2)
Liver cirrhosis	179	(0.2)	179	(0.2)
Chronic kidney disease	440	(0.4)	440	(0.4)
Asthma	2036	(1.8)	2036	(1.8)
Stroke	798	(0.7)	798	(0.7)
Cancer	2049	(1.8)	2049	(1.8)
Osteoporosis	704	(0.6)	704	(0.6)
Alcohol-related illness	577	(0.5)	577	(0.5)
Parkinson's disease	815	(0.7)	815	(0.7)
Number of hospitalizations†				
0	104468	(91.9)	104468	(91.9)
1	7788	(6.9)	7788	(6.9)
2	931	(0.8)	931	(0.8)
≥3	438	(0.4)	438	(0.4)
Number of emergency visits†				
0	74908	(65.9)	74908	(65.9)
1	28007	(24.7)	28007	(24.7)
2	7536	(6.6)	7536	(6.6)
≥3	3174	(2.8)	3174	(2.8)

COPD, chronic obstructive pulmonary disease.

*After matching by propensity score, there is no patients in other ill-defined fractures of trunk, other upper limb, and fracture of unspecified bones.

†Before fracture admission within one year

Compared with women (after matching procedure in Table 3), men had higher risks of post-fracture mortality (OR1.40, 95% CI 1.19–1.64) and complications, including pneumonia (OR 1.77, 95% CI 1.62–1.93), acute renal failure (OR 1.60, 95% CI 1.33–1.91), deep wound infection (OR 1.60, 95% CI 1.47–1.75), stroke (OR 1.59, 95% CI 1.49–1.70), septicemia (OR 1.53, 95% CI 1.42–1.64) and acute myocardial infarction (OR 1.57, 95% CI 1.16–2.12). Longer length of hospital stays (6.6±6.3 vs. 6.2±5.7 days, p < 0.0001) and increased medical expenditures (1743±1797 vs. 1672±1589 US dollars, p < 0.0001) were also noted in the male patients. However, lower risk of urinary tract infection after fracture was found in men compared with women (OR 0.64, 95% CI 0.60–0.68).

**Table 3:** Risk of complications and mortality after fracture for female and male patients.

	Female (n = 100864)		Male (n = 100864)		Risk of outcomes	
	Events	%	Events	%	OR	(95% CI)*
30-day in-hospital mortality	278	0.3	381	0.4	1.40	(1.19–1.64)
Postoperative complications						
Pneumonia	851	0.8	1456	1.4	1.77	(1.62–1.93)
Acute renal failure	198	0.2	314	0.3	1.60	(1.33–1.91)
Deep wound infection	836	0.8	1329	1.3	1.60	(1.47–1.75)
Stroke	1648	1.6	2514	2.5	1.59	(1.49–1.70)
Septicemia	1198	1.2	1811	1.8	1.53	(1.42–1.64)
Acute myocardial infarction	70	0.1	109	0.1	1.57	(1.16–2.12)
Postoperative bleeding	149	0.2	171	0.2	1.15	(0.92–1.43)
Pulmonary embolism	65	0.1	74	0.1	1.14	(0.81–1.59)
Urinary tract infection	2826	2.8	1870	1.9	0.64	(0.60–0.68)
Medical expenditure, US dollars†	1672±1589		1743±1797		p<0.0001	
Length of hospital stay, days†	6.2±5.7		6.6±6.3		p<0.0001	

CI, confidence interval; OR, odds ratio.

*Adjusted for all covariates listed in Table 2.

†Mean±standard deviation. For the medical expenditure, the median and interquartile range were 1328 (954–2073) in men and 1308 (950–2051) in women; For the length of hospital stay, the median and interquartile range were 5 (3–8) in men and 5 (3–7) in women.

In Table 4, the association between male gender and the risk of post-fracture adverse events was significant in patients aged 40–49 years (OR 1.20, 95% CI 1.07–1.34), 50–59 years (OR 1.23, 95% CI 1.13–1.34), 60–69 years (OR 1.38, 95% CI 1.25–1.51), 70–79 years (OR 1.39, 95% CI 1.29–1.51), and ≥80 years (OR 1.69, 95% CI 1.58–1.81). Male gender was associated with increased risk of adverse events after fracture in patients with zero (OR 1.24, 95% CI 1.17–1.32), one (OR 1.25, 95% CI 1.17–1.34), two (OR 1.24, 95% CI 1.14–1.33), more than three (OR 1.19, 95% CI 1.10–1.28) documented medical conditions. Post-fracture adverse events were associated with male gender for patients with various subtypes of fracture.

**Table 4:** The stratified analysis for the risk of adverse events after fracture associated with sex.

			Adverse events*			
		n	Events	Rate, %	OR	(95% CI)†
Age 20–29 years	Female	14965	536	3.6	1.00	(reference)
	Male	14965	564	3.8	1.06	(0.93–1.20)
Age 30–39 years	Female	12206	454	3.7	1.00	(reference)
	Male	12206	461	3.8	1.02	(0.89–1.17)
Age 40–49 years	Female	15635	595	3.8	1.00	(reference)
	Male	15635	699	4.5	1.20	(1.07–1.34)
Age 50–59 years	Female	24800	1113	4.5	1.00	(reference)
	Male	24800	1342	5.4	1.23	(1.13–1.34)
Age 60–69 years	Female	12237	854	7.0	1.00	(reference)
	Male	12237	1124	9.2	1.38	(1.25–1.51)
Age 70–79 years	Female	10112	1270	12.6	1.00	(reference)
	Male	10112	1668	16.5	1.39	(1.29–1.51)
Age ≥80 years	Female	10909	2169	19.9	1.00	(reference)
	Male	10909	2471	22.7	1.18	(1.11–1.26)
0 medical condition	Female	55426	2250	4.1	1.00	(reference)
	Male	55426	2725	4.9	1.24	(1.17–1.32)
1 medical condition	Female	21953	1789	8.2	1.00	(reference)
	Male	21953	2156	9.8	1.25	(1.17–1.34)
2 medical conditions	Female	13121	1467	11.2	1.00	(reference)
	Male	13121	1743	13.3	1.24	(1.14–1.33)
≥3 medical conditions	Female	10364	1485	14.3	1.00	(reference)
	Male	10364	1705	16.5	1.19	(1.10–1.28)
Fracture of skull	Female	1391	526	37.8	1.00	(reference)
	Male	1391	512	36.8	0.96	(0.82–1.12)
Fracture of face bones	Female	4872	99	2.0	1.00	(reference)
	Male	4872	146	3.0	1.50	(1.15–1.94)
Other skull fracture	Female	281	110	39.2	1.00	(reference)
	Male	281	118	42.0	1.14	(0.80–1.62)
Vertebral column	Female	3093	311	10.1	1.00	(reference)
	Male	3093	426	13.8	1.44	(1.23–1.68)
Rib, sternum, larynx and trachea	Female	684	49	7.2	1.00	(reference)
	Male	684	72	10.5	1.56	(1.06–2.31)
Pelvis	Female	544	65	12.0	1.00	(reference)
	Male	544	72	13.2	1.13	(0.78–1.64)
Clavicle or scapula	Female	9420	193	2.1	1.00	(reference)
	Male	9420	200	2.1	1.04	(0.85–1.27)
Humerus, radius or ulna	Female	25991	886	3.4	1.00	(reference)
	Male	25991	1072	4.1	1.22	(1.12–1.34)
Carpal, metacarpal, or phalanges	Female	4962	152	3.1	1.00	(reference)
	Male	4962	137	2.8	0.90	(0.71–1.14)
Fracture of femur	Female	19281	3187	16.5	1.00	(reference)
	Male	19281	3847	20.0	1.27	(1.20–1.34)
Patella, tibia, or fibula	Female	14702	772	5.3	1.00	(reference)
	Male	14702	1037	7.1	1.37	(1.25–1.51)
Ankle, tarsal and metatarsal bones, phalanges of foot	Female	15583	640	4.1	1.00	(reference)
	Male	15583	687	4.4	1.08	(0.97–1.20)
Multiple fractures of upper limbs, lower limbs or trunk	Female	59	1	1.7	1.00	(reference)
	Male	59	3	5.1	3.49	(0.32–38.4)
Fracture without surgery	Female	20920	2512	12.0	1.00	(reference)
	Male	20920	2974	14.2	1.24	(1.17–1.32)
Fracture with surgery	Female	79944	4479	5.6	1.00	(reference)
	Male	79944	5355	6.7	1.23	(1.18–1.28)

CI, confidence interval; OR, odds ratio.

*Adverse events included with 30-day in-hospital mortality, stroke, pneumonia, septicemia, acute renal failure, acute myocardial infarction, deep wound infection, postoperative bleeding, pulmonary embolism, urinary tract infection.

†Adjusted for all covariates listed in Table 2.

## Discussion

This nationwide, propensity score-matched, retrospective population-based study showed that male gender was independently associated with higher post-fracture 30-day in-hospital mortality, complications (including stroke, acute renal failure, and acute myocardial infarction), and infectious conditions (such as pneumonia, septicemia and deep wound infection). Increased medical expenditures and longer length of hospital stays were also noted in males compared to females.

Several previous investigations have tried to determine the association between sex differences and post-fracture mortality rates. Most studies reported a higher long-term (>120 days) post-fracture mortality rate among men [6,8,9,20,21,22], which is consistent with our study result. Our study showed that male patients have higher 30-day in-hospital mortality rate, compared with female patients. We analyzed 30-day in-hospital mortality, a relatively short-term cut point, instead of a long-term cut point as previous researchers did in order to further reinforce the relationship between fractures and mortality.

Several studies suggested male gender as a risk factor for major infection in post-fracture and trauma populations [23,24], and more male patients suffer from severe sepsis or septic shock in surgical intensive care units [25]. Similar to previous studies, we noted that male patients had higher incidences of post-fracture pneumonia, deep wound infections, and septicemia, while female patients had a higher incidence of urinary tract infections. Mounting evidence in both animal and human studies showed that sex hormones play a role in immunomodulation, which could affect the incidence and severity of infections [26–30], suggesting a reasonable explanation as to why female patients may tolerate sepsis better. Splenocyte proliferative capacity and splenocyte interlukin-2 and interlukin-3 responses were remarkably decreased in male septic mice, leading to decreased survival rates [27]. Human studies showed higher levels of a pro-inflammatory mediator (tumor necrosis factor alpha) in men than women, and lower levels of an anti-inflammatory mediator (interlukin-10) in women [29], demonstrating the different immune reactions between genders during sepsis.

In our study, post-fracture infections may account for longer hospital stays, higher medical expenditures, and higher mortality rates. One research has demonstrated that post-hip fracture chest infection may contribute to 30-day mortality, while deep wound infection and urinary tract infection did not [31]. Another study using adjusted logistic regression model analysis concluded that mortality rates were similar between males and females after excluding deaths caused by infections [23]. The fact that men and women have different incidences and severities of infectious diseases should be of greater clinical importance, as it may affect the prognosis significantly.

Post-fracture stroke rate was noted to be higher in men than women. According to previous research, the incidence of stroke is lower in women who are less than 85 years of age, and the first stroke event tends to occur at an older age [32,33]. This may be explained by the neuroprotective effects of estrogen and progesterone [34–36]. Estrogen mediates its neuroprotective effect through several mechanisms, including its antioxidant effect, the preservation of autoregulation functions, and decreased cell apoptosis. Progesterone has an effect on membrane stabilization and decreasing cell excitotoxicity through glutamate receptor inhibition [34]. The neuronal preservation tends to be better in women than men after ischemic events [35,36].

The data also showed higher acute myocardial infarction rates in men than women. Estrogen is known to have a cardioprotective effect through various mechanisms [37], thus it is not surprising that previous research has already revealed a gender gap in the risk of myocardial infarction and other cardiovascular diseases [38–40]. The incidence of acute renal failure is also higher in men. However, we were unable to obtain information about pre-fracture renal function, medications, and fluid administration via our database, so careful interpretation of this finding is warranted.

Longer hospital stays and increased medical expenditures in male patients were likely due to them experiencing a greater number of adverse events. Other factors that have post-fracture gender differences and might have influenced post-fracture hospital stays and expenses include post-fracture functional status, rehabilitation conditions, and the patients’ socioeconomic support [41–44]. Future studies may focus on post-fracture care since this issue may be important to integrated care and cost-savings.

We performed subgroup analyses, which were stratified according to age, number of medical conditions and previous fracture locations. The rate of adverse events shows significant differences among age groups, and the differences tend to be greater as age increases. Additionally, this gender difference is still prominent after accounting for the number of medical conditions and different fracture locations. In addition, we also observed that the gender difference exists in both fracture patients who underwent orthopedic surgery and those who did not. However, this phenomenon was first reported in the present study.

Our study has some limitations. First, our database lacks information about patients’ sociodemographics and lifestyles, as well as their detailed clinical conditions, including pre-fracture and post-fracture physical function, perioperative findings and in-hospital laboratory examinations. Second, we used claimed ICD-9-CM codes, which could not demonstrate the severity of comorbid illnesses, or the mechanism of the injuries. Third, the validation of Taiwan’s National Health Insurance Research Database remains inadequate although the physician’s diagnosis and codes of diseases were validated in previous studies [45–51]. Fourth, the information of diagnosis-related group and self-payment was not available in the insurance database in Taiwan. It is also a limitation that we could not evaluate the influence of diagnosis-related group and expensive self-payment orthopedic instrument on the medical expense and length of hospital stay. In addition, the American Society of Anesthesiologists grade is a good indicator for determining general health status. However, it is not available in Taiwan’s National Insurance Research Database. Finally, although we performed propensity score matching and multivariate adjustment, some residual confounding bias remains possible. There were many potential confounding factors were not considered in this study.

In conclusion, we raised the possibility that men had more complications and higher mortality than women, with the exception of increased rates of urinary tract infections in women. This phenomenon could be observed in the subgroup analysis. However, the interpretation of our findings should be cautioned because of several study limitation.

## List of abbreviations

ICD-9-CM: International Classification of Diseases, 9th Revision, Clinical Modification
CI: confidence interval
OR: odds ratio
