# Correction: Sex differences in fracture outcomes within Taiwan population: A nationwide matched study

**DOI:** 10.1371/journal.pone.0234229

**Published:** 2020-05-29

**Authors:** Fang-Pai Chou, Hung-Chi Chang, Chun-Chieh Yeh, Chih-Hsing Wu, Yih-Giun Cherng, Ta-Liang Chen, Chien-Chang Liao

The last two authors, Ta-Liang Chen and Chien-Chang Liao, should be noted as contributing equally to this work.
